# 3D-printing a cost-effective model for mastoidectomy training

**DOI:** 10.1186/s41205-023-00174-y

**Published:** 2023-04-17

**Authors:** Andreas Frithioff, Kenneth Weiss, Martin Frendø, Pascal Senn, Peter Trier Mikkelsen, Daniel Sieber, Mads Sølvsten Sørensen, David Bue Pedersen, Steven Arild Wuyts Andersen

**Affiliations:** 1 Copenhagen Hearing and Balance Center, Dept. of Otorhinolaryngology—Head & Neck Surgery and Audiology, Rigshospitalet, Copenhagen, Denmark; 2 Department of Mechanical Engineering, Technical University of Denmark, Kgs. Lyngby, Denmark; 3 Copenhagen Academy for Medical Education and Simulation (CAMES), Center for HR & Education, Region H, Copenhagen, Denmark; 4 Department of Plastic Surgery, Herlev & Gentofte Hospital, Copenhagen, Denmark; 5 Department of Clinical Neurosciences, Service of ORL & Head and Neck Surgery, University Hospital of Geneva, Geneva, Switzerland; 6 Department of Medical & Health Technologies, MCI | The Entrepreneurial School, Innsbruck, Austria; 7 Institute for Clinical Medicine, University of Copenhagen, Copenhagen, Denmark

**Keywords:** 3D printing, Additive manufacturing, Rapid prototyping, Temporal bone, Mastoidectomy, Training, Education, Otology

## Abstract

**Background:**

3D-printed temporal bone models can potentially provide a cost-effective alternative to cadaver surgery that can be manufactured locally at the training department. The objective of this study was to create a cost-effective 3D-printed model suitable for mastoidectomy training using entry level and commercially available print technologies, enabling individuals, without prior experience on 3D-printing, to manufacture their own models for basic temporal bone training.

**Methods:**

Expert technical professionals and an experienced otosurgeon identified the best material for replicating the temporal bone and created a cost-effective printing routine for the model using entry-level print technologies. Eleven participants at a temporal bone dissection course evaluated the model using a questionnaire.

**Results:**

The 3D-printed temporal bone model was printed using a material extrusion 3D-printer with a heat resistant filament, reducing melting during drilling. After printing, a few simple post-processing steps were designed to replicate the dura, sigmoid sinus and facial nerve. Modifying the 3D-printer by installing a direct-drive and ruby nozzle resulted in more successful prints and less need for maintenance. Upon evaluation by otorhinolaryngology trainees, unanimous feedback was that the model provided a good introduction to the mastoidectomy procedure, and supplementing practice to cadaveric temporal bones.

**Conclusion:**

In-house production of a cost-effective 3D-printed model for temporal bone training is feasible and enables training institutions to manufacture their own models. Further, this work demonstrates the feasibility of creating new temporal bone models with anatomical variation to provide ample training opportunity.

**Supplementary Information:**

The online version contains supplementary material available at 10.1186/s41205-023-00174-y.

## Introduction

Cadaveric temporal bones remain the gold standard for learning temporal bone surgery, where high-quality training is imperative for ensuring patient safety. Nonetheless, limited availability of cadavers for training combined with a high demand for high-quality training have increased interest in alternatives to cadaveric dissection[1–4]. 3D-printing (also called additive manufacturing) is a set of promising technologies, which have been proposed for producing physical models for simulation-based training of temporal bone surgery [4–6].

Despite a general assumption that 3D-printed models hold great potential in the context of surgical training in otology, systematic implementation into surgical curricula is scarce, and evidence on educational effectiveness limited [2, 5, 7]. Only recently has it been documented that training mastoidectomy on 3D-printed models actually improves novices’ surgical performance during subsequent cadaveric dissection (i.e., transfer of skills) [8, 9]. However, poor physical resemblance and high costs are substantial barriers to implementation [10].

Several studies aiming to validate 3D-printed temporal bone models exist, but none have thus far provided a thorough reporting on the process behind 3D-printing the models [10]. This is a problem because potential user benefit requires sufficient technical information for peer replication. The term 3D-printing refers to various processes and choosing the right print-technology, print settings and material is crucial for the outcome. 3D-printed models can potentially create an inexpensive, effective and practical alternative to cadaver surgery for training departments and increases the number of temporal bones available for trainees to practice repeatedly to proficiency. Nevertheless, for training departments to be able to 3D-print their own temporal bone models a detailed technical description of the manufacturing process is required. In this study, designed in conjunction between experienced otologists, 3D-print experts and educational scientists, we aim to create a cost-effective, high-fidelity 3D-printed temporal bone model using entry-level and commercially available print technologies. Further, we will provide directions and key points on the development and manufacturing processes, including software recommendations, relevant printing equipment, and necessary steps during design and production of the model. This will enable other training departments, without engineering capacities or prior 3D-printing experience, to start manufacturing their own 3D-printed models for temporal bone training. We further aim to evaluate the perceived usefulness of the model prior to cadaver surgery during a temporal bone course for residents in otorhinolaryngology (ORL).

## Materials and methods

### Creating a printable file

**Fig. 1:**
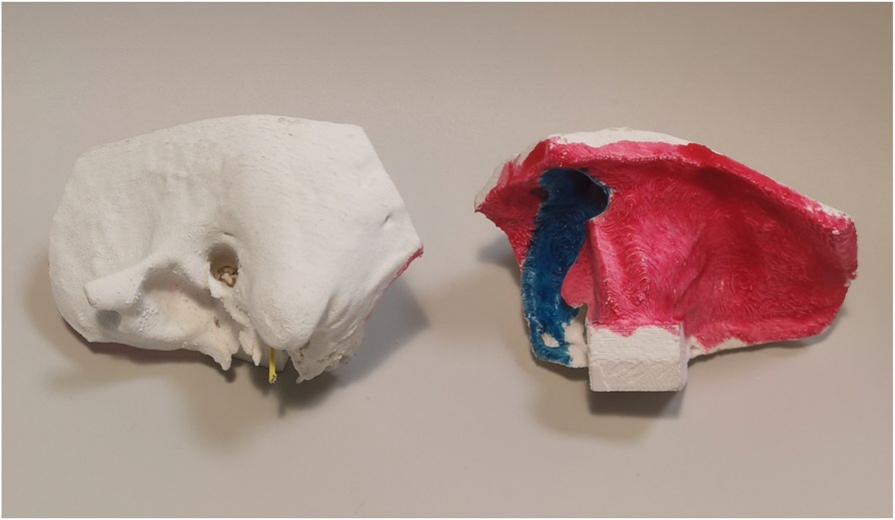
The 3D-printed Temporal Bone model. Left side shows the lateral/superficial part of the temporal bone model; right side shows the medial side (red representing dura mater and blue representing the sigmoid sinus)

The model is based on cone-beam computed tomography scans (CBCT) from the publicly available OpenEar library [11]. The OpenEar library comprises eight digitized and segmented temporal bone models with 3D reconstruction (included in the folder “3D models” [11]). The folder contains Polygon (PLY) files of the bony volume, inner ear cavompartments, the ossicles, tympanic membrane, nerves and vessels. We used the dataset “Delta” and the PLY files “Bone”, “Malleus”, “Incus”, “Stapes”. The file “Bone” was imported to the freeware Meshmixer (https://www.meshmixer.com/); the auto repair function was used to optimize the bone. Next, the files “Malleus”, “Incus” and “Stapes” were imported and the four files merged with the “combine” function and exported as a Standard Triangle (STL) file. In order to fixate the model during drilling, we added a block on the back of the model using SolidWorks (Dassault Systemes, Vélizy-Villacoublay, France; Fig. 1), however this step can also be performed using Meshmixer. The print job generation was carried out in Ultimaker Cura freeware (Ultimaker, Utrecht, Netherlands) (https://ultimaker.com/software/ultimaker-cura), orienting the model such that the important areas were orthogonal to the build plane, and facing upwards. The Ultimaker Cura software translates the STL-file to a G-code which is the final file format that the 3D-printer uses for printing the model. Another freeware version of the Cura software dedicated for use with the Creality Ender 3 Pro printer is available for download as Creality Slicer (https://www.creality.com/download).

### Printing-material

To determine the best material for replicating bone properties, while also remaining cost-effective and easy to use with extrusion printers, two experts on 3D-printing (DBP & KW) identified eight different materials for testing. The materials included different plastics loaded with various fillers to make them more heat resistant to avoid agglomerating when drilling. We printed a sample (a cube of 5 × 5x5cm) of each material and a senior otologist with extensive experience in temporal bone surgery (MSS) drilled the eight different materials with both sharp and diamond drills to determine which material best represented temporal bone properties. After identifying the most suitable material from the samples we printed a complete temporal bone model which were drilled by the senior otologist.

### 3D-printer & calibration

**Fig. 2:**
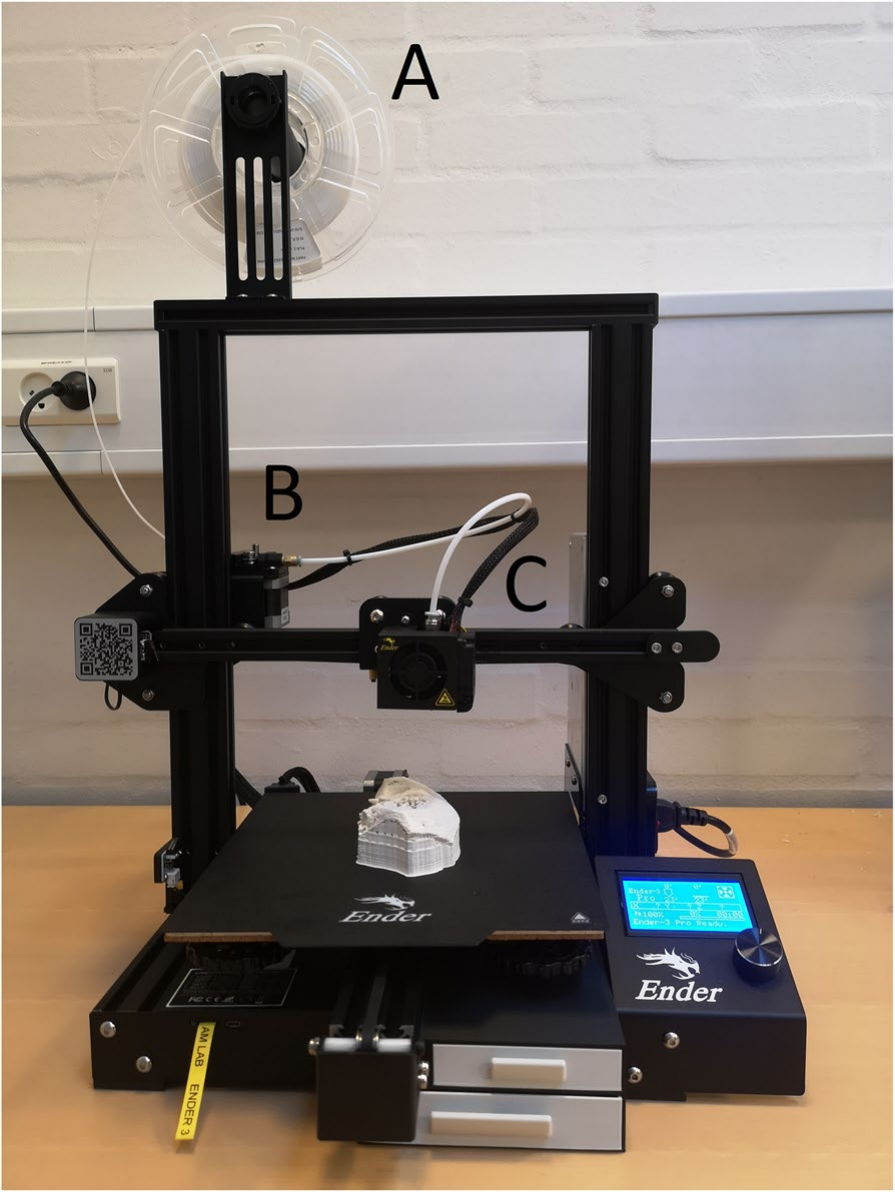
Material Extrusion based 3D-printer (Ender-3, Creality). **A** Filament, **B** Driver gear moving the filament through the white Bowden tube to the hot end and **C** Nozzle extruding the material

The printing principle of material extrusion entails advancement of thermoplastic filament into a heated extruder barrel and exit through a nozzle whereby strands of molten plastic deposit. The object is thus built in a layer-by-layer manner (Fig. 2). We chose a material extrusion 3D-printer (Ender-3 Pro, Creality, Shenzhen, China). This printer is commercially available and inexpensive (~ 300 USD).

There are certain parameters that can be controlled in the print process such as the printing speed, temperature of the extruder and printing bed, and the infill of material to the nozzle. All these parameters affect the quality of the print. To determine the optimal printing settings with the chosen material, we first printed a temperature calibration tower, which is designed to test the formation on different shapes at different temperature levels (https://www.thingiverse.com/thing:2729076). After determining the optimal printing temperature, we optimized the other parameters iteratively in printing temporal bone models.

### Evaluation of the model for training

Eleven otorhinolaryngology (ORL) residents and attending physicians participated in a temporal bone dissection course at University Hospital of Geneva, Geneva, Switzerland. The course was a 2-day practical course. The first day, the participants performed 2–3 procedures on the Visible Ear Simulator—a virtual reality temporal bone surgical simulator (https://ves.alexandra.dk/) and two anatomical mastoidectomies on the described 3D-printed. The following day, participants performed two mastoidectomies on a human cadaver (one on each side) and immediately after this, completed a questionnaire on their experience with drilling the 3D-printed models compared with cadaver surgery. The questionnaire consisted of two parts: A) the participant’s opinion on the model as an addition to cadaver surgery (Table 1), and B) the participant’s background data such as their experience. In part A, participants were asked to which extent they agreed with five statements concerning the model. For example, participants should state whether it was helpful to drill the 3D-printed model prior to cadaver surgery and how the physical properties of the model compared with drilling the human temporal bone.

**Table 1:** Estimated manufacturing cost per model

Wearables and consumptions	Material cost (USD)	Models printed before needing replacement (n)	Cost per model (USD)
Material (Filament, latex, wire and glue)	5	1	5
Direct drive	78	250	0.3
Bowden tube	4	250	0.016
Ruby nozzle	60	100	0.6
Lab technician	9 (per 20 min.)	n/a	9
Power consumption	0.2 (per 1 kWh)	n/a	0.2
3D-printer	300	250 per year in 5 years	0.25

## Results

### The Printable file

The STL-file and the G-code of the model can be downloaded freely from the internet by using the following link: https://www.rigshospitalet.dk/temporal-bone-imaging-and-simulation-research-group.

### 3D-printing material

Melting during drilling and creation long of shavings was a major problem in seven of the eight materials (Additional file 1). The best material for printing the temporal bone model was found to be the Lay-brick (CC-products, Cologne, Germany), a filament which is a milled chalk composite in a matrix of polylactic acid (PLA). The high content of finely milled chalk prevents the material from agglomerating and melting when drilling. This is crucial during drilling exercises since regular PLA based filaments liquify from the heating caused by drilling friction, thus agglomerating and packing in the flutes of the drill bit. This makes regular PLA unfit to replicate bone interaction with the drill bit and therefore for use for simulation-based training. However, the high load of chalk filler also brittles the Lay-brick filament, which initially resulted in the risk of filament breakage during printing, causing unsuccessful prints. Finally, the chalk filler makes the filament slightly abrasive, resulting in both the nozzle and the drive gear (i.e., the part of the printer feeding the nozzle with filament; Fig. 2) to wear out faster than usual. This results in the standard nozzle needing replacement after approximately printing five models and the drive gear twenty models.

### 3D-printer

**Fig. 3:**
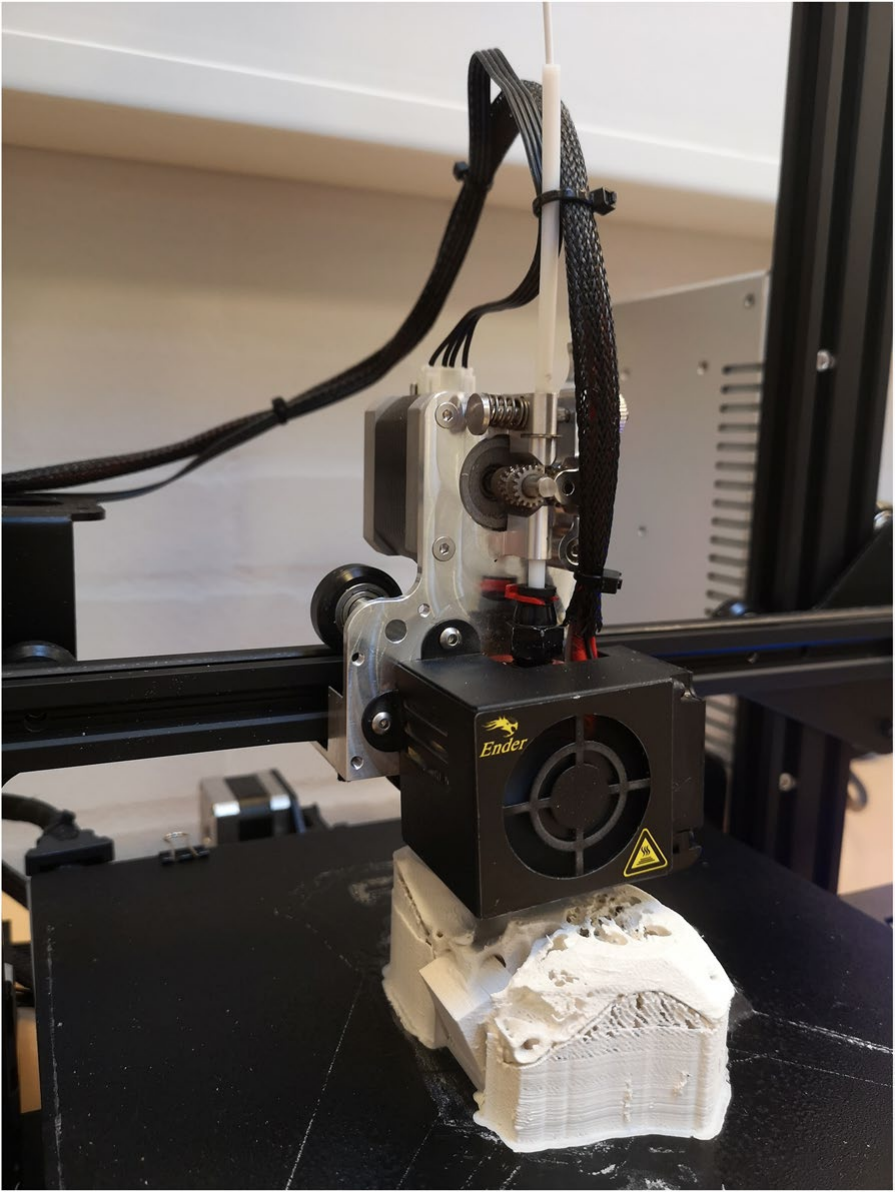
Direct-filament drive installed on Material Extrusion based 3D-printer (Ender-3, Creality)

To avoid the filament from breaking and to extend the durability of the service parts, we made some minor modifications to the printer. First, we installed a direct filament drive (Micro Swiss Direct Drive Extruder for Creality Ender3, Mico Swiss, Ramsey, MN 5303 USA), which cost ~ 80 USD. Using a direct filament drive, the drive gear is fitted directly on the hot end instead of being guided through a Bowden tube (Fig. 3) to avoid tension from building up in the Bowden tube between the drive gear and nozzle, which can otherwise cause the filament to break. Also, the direct drive is made of steel instead of brass which reduces wearing. Furthermore, we replaced the standard nozzle with a more durable Ruby nozzle (PrimaCreator MK8, Ruby Nozzle 0.4 mm, PrimaCreator, Malmö, Sweden, ~ 60USD) making it possible to print more than 100 models before nozzle replacement. The total cost of the printer including modifications was ~ 430 USD. The Lay-brick filament is prone to build-up of charred residuals, which can result in clogging of the nozzle if it is not regularly cleaned by reaming the nozzle with a piano-wire (Ø = 0.4 mm). Lastly, the filament was stored on a wheel/barrel with a larger diameter and fitted on a stand, ensuring a more direct infeed to the direct drive of the printer.

For the optimal print, the printing speed was found to be 50 mm/s, infill 98% and the temperature of the extruder and printing bed 195ºC and 35ºC, respectively. With these printer-settings it took approximately 16 h to complete the printing of a single model, requiring 65 g of material.

### Post-processing of the print

After 3D-printing, the model underwent several post-processing (i.e., post-printing) steps before it was suitable for training. To support overhanging structures during printing, a low-density support-structure needs to be printed and manually removed after printing. Further, non-bony structures such as the dura, sigmoid sinus and facial nerve need to be added after printing. We represented the dura and sigmoid sinus by applying a latex layer mixed with either purple or blue color to the relevant areas using a sponge brush and the facial nerve by placing a piece of thin electrical wire with a yellow jacket in the fallopian canal (Fig. 1).

### Manufacturing cost

Printer start-up and model post-processing was handled by a laboratory technician in our research lab during the daily routines, with printer start-up during work hours and postprocessing the next morning*.* The start-up and post-processing took approximately 20 min per model including: Removing the model from the printer, removing support structures, inserting the facial nerve and coloring the model. The total material cost including, filament, latex and wire was ~ 5 USD. We estimate that the bowden tube and direct drive needs replacement after printing approximately 250 models. With an estimated power consumption of ~ 0.1kWh per print and deprecation of the printer over 5 years, printing 250 models a year, the total manufacturing cost per model is 15.4 USD (Table 1).

### Drilling

When using the model for drilling exercises, we found that several factors should be considered: First, even though the Lay-brick filament’s better resistance to melting from thermal friction than other thermoplastics, minor melting and agglomeration still occurred when drilling the model. To avoid this, we recommend only using sharp burrs as this causes less melting of the model than diamond burrs.

Further, irrigation should be avoided as it leads to clustering of tiny fragments of plastic. When using suction, we found it necessary to use a suction tip with a large diameter as the drilling fragments are larger than those occurring when drilling real bone. However, even when using a large suction tip, clogging occurred occasionally, and the best way to avoid this is either to turn the model upside down or use compressed air to remove plastic fragments from the model during drilling.

### Model evaluation

Eleven ORL residents and attending physicians completed the questionnaire after drilling exercises. The participants had between 1 and 8 years of ORL experience and most had limited experience with the mastoidectomy procedure (0–3 performed mastoidectomies), with a single participant having performed 20 mastoidectomies.

Overall, participants evaluated the model positively on all domains (Table 2). All participants agreed or strongly agreed that the model served as a good introduction to the procedure and increased the understanding on the procedural steps. All participants also strongly agreed/agreed that the anatomical representation was accurate and the majority (81%) strongly agreed/agreed that the feeling when drilling was bone-like. Only two participants (19%) disagreed with the statement that the plastic of the model was comparable to a real bone.

**Table 2:** Evaluation of the model

Question	Strongly agree	Agree	Neither agree or disagree	Disagree	Strongly disagree
*Using the 3D-printed models was helpful learning to use the surgical microscope and drills before proceeding to cadaver surgery*	21% (9)	19% (2)	0% (0)	0% (0)	0% (0)
*Drilling the 3D-printed models provided a better understanding of the mastoidectomy procedure before performing cadaver surgery*	73% (8)	27% (3)	0% (0)	0% (0)	0% (0)
*The anatomy of the model was comparable to a real temporal bone, excluding soft tissue components*	9% (1)	91% (10)	0% (0)	0% (0)	0% (0)
*The plastic of the model was comparable to drilling the real bone*	19% (2)	62% (7)	0% (0)	19% (2)	0% (0)
*The 3D-printed model served as a good introduction to the cadaver surgery*	91% (10)	9% (1)	0% (0)	0% (0)	0% (0)

## Discussion

In this study, we present the manufacturing of a cost-effective 3D-printed temporal bone model, providing sufficient details for in-house replication in ORL training departments. In addition, we evaluate ORL trainees’ opinion of the model’s utility during a temporal bone dissection course. The model was created using an entry level and commercially available 3D-printer, which can be acquired for ~ 430 USD including installing a direct drive and ruby nozzle. Each model printed had a material cost of ~ 5 USD and an estimated total manufacturing cost of 15.4 USD. This enables most departments to start in-house production of 3D-printed models, providing an effective and inexpensive alternative to commercially available models. Further, participants at a temporal bone course unanimously agreed that the model serves as a good introduction to the mastoidectomy procedure.

Even though 3D-printed models are perceived to have an important role in the future temporal bone training curriculum, [4] it should be noted that 3D-printing is not just “plug-and-play”. Considerations on the material and maintenance of the printer is essential. We chose to use material extrusion-based 3D-printing technology as it is inexpensive, easy to use, and effectively reproduces the air cells in the mastoid bone without residual materials being trapped during the print process. This is in contrast to other more advanced and expensive print technologies where unconsolidated residual material easily entraps in the air cells [10, 12]. To avoid melting and agglomerating when drilling, we chose a filament with a high load of chalk filler. However, this filament is very fragile and easily breaks during printing. To avoid this, we made some minor modifications to the printer such as installing a direct filament drive and replacing the standard nozzle with a so-called Ruby nozzle (e.g. PrimaCreator MK8, Ruby Nozzle 0.4 mm, PrimaCreator, Malmö, Sweden, ~ 60USD). These modifications easy to perform, reduces the need for maintenance and ensures high quality of the printing. The Ruby nozzle has a ruby gemstone at the tip of the nozzle enhancing durability: A standard nozzle (~ 5 USD) gets worn out after approximately five prints whereas the Ruby nozzle (~ 60 USD) can last more than 100 prints, reducing the need for replacement and maintenance. Installing the direct filament drive, lowering the risk for failed prints due to the filament breaking, is easy, takes 1–1.5 h and does not require major printer modifications. Alternatively, investing in a printer with direct drive installed as a standard (e.g., the ~ 1,000 USD Prusa i3 MKS3 + , Prusa Research, Czech Republic *or* the ~ 550 USD Ender-3 Pro S1, Creality, Shenzhen, China) would remedy this issue. However, the design of the Prusa i3’s and Ender-3 Pro S1’s hot end is more compact and complicates cleaning and maintenance. Although any of the suggested printers, without any modifications, is suitable for printing temporal bone models, we recommend using the Ender-3 Pro with the suggested modifications (total price of ~ 430 USD) for the lowest cost, lowest need for maintenance, and highest rate of successful prints compared with out-of-the box 3D-printers. Setting up the printer and installing the suggested modifications does not require professional engineering support, and can potentially be done by any individual with the interest of 3D-printing the temporal bone model presented in this paper.

While the presented model closely replicates temporal bone properties, post-processing is necessary to represent important soft-tissue structures. Consequently, the dura and sigmoid sinus are represented by colored latex layers and the facial nerve by a yellow wire. In the current iteration of the model, the chorda is not represented and the lateral semicircular canal and ossicles are printed in the same material as the remaining bony parts and therefore lack specific visual cues. The ossicles are inadequately replicated due to the print resolution limitations; more accurate representation would require a higher resolution printer [13]. When performing a mastoidectomy in the OR, surgeons depend on identifying key anatomical structures and using those visual cues to perform a safe procedure [14]. In addition to solving these problems, reducing the need for post-processing steps would enable mass-production. This would likely require the use of more advanced and costly print technologies. Further, even with the model being printed using a heat resistant material, there are still some issues with melting when drilling in the same spot for too long. Also, drilling fragments are larger than those occurring when drilling real bone meaning that the suction easily clots. This could also explain why two participants on the questionnaire disagreed with the plastic being comparable to drilling real bone. Nevertheless, the majority of the participants found the drilling of the model and plastic comparable with a real cadaveric temporal bone.

Other studies have evaluated the most suitable material for replicating drilling of the bone [4, 15–17] and found resin to best mimic the hardness of the bone. However, this material requires using the more expensive photopolymerization printers and would increase the price per model, including the demand for manual post-processing as liquid residual material is entrapped in the mastoid air cells [12]. Even though the Lay-brick filament is more heat resistant compared to other PLA materials still some melting occurs when drilling, especially when using a diamond drill. We therefore recommend only using sharp drills as this seems to limit this problem. Another solution could be to find an even more heat resistant filament that could be used in an extrusion-based printer. However, this would likely complicate the printing process as the material would be even more brittle compared with the Lay-Brick filament. Correspondingly, it is imperative to find a balance between the best possible drilling material and feasibility during the printing process.

The participants at the temporal bone drilling course rated the model as being very useful prior to cadaveric training. While helpful, such opinions do not document or “validate” that the 3D-printed model is indeed a good and effective training tool for novice surgeons acquiring actual surgical skills. Repeatedly it has been demonstrated that such subjective outcomes are poorly correlated with actual surgical skills [18–20] and establishing the educational value (i.e. whether the trainees actual gain surgical skills) should rely on objective performance outcomes using established assessment tools [18]. Recently, we collected such validity evidence supporting that training using 3D-printed temporal bone models improve trainees’ subsequent cadaver dissection performance [9]. Knowing that trainees actually benefit from the specific educational intervention warrants using trainee opinions as an outcome in the present study. The positive attitude for using the 3D-printed models prior to cadaver surgery reflects the trainees being motivated for using this new training intervention, which is important for implementation of 3D-printed models in the training curricula.

A strength of our approach to developing the model is drawing on multidisciplinary expertise in 3D-printing, surgical otology, and surgical educational research to ensure methodical rigor. To obtain detached views, we collected data from residents from another institution and country than the developers of the model. This reduces the risk of bias compared to evaluation at the home institution. Finally, the 3D-printed temporal bone model described is derived from freely available data, and has undergone collection of educational validity evidence (“validation”) according to modern educational methodology [9]. Nonetheless, despite the positive attitude towards the 3D-printed temporal bone models, this was a single-site evaluation with a relatively small sample size representing a limitation of this study. Another limitation is that the cost analysis was partly based on estimates instead of actual observations. The life-span of wearables are highly dependent on several factors such as: The technical knowhow of the operator, the frequency of use, what is being printed and ongoing maintenance. Printing routines by non-technical staff is still new at our institution and consequently, the life-span of the direct-drive and bowden tube is based on estimates made by technical professionals. Despite these uncertainties, the cost analysis still offers important knowledge for institutions planning to start their own 3D-printing routines as depreciation of hardware and human resources represents a notable additional cost [21].

Altogether, the presented workflow is suited for a relatively small in-house production (for example larger training centers) and we find that in-house production of an inexpensive and effective 3D-printed model for temporal bone training is feasible. We have provided both STL-files and G-codes which together with the technical descriptions provided in this paper, enables other ORL colleagues, without prior knowledge on 3D-printing, to start manufacturing their own models to provide ample training opportunity for their training institution. Furthermore, the OpenEar library consists of seven additional segmentations of temporal bones which can be translated into printable files, potentially creating opportunity to train on different anatomies. Future work aims to create an even more accurate drilling experience, optimizing the workflow, to create different anatomical models and performing further validation of the 3D-printed models as a tool for temporal bone training.

## Conclusion

Using an entry level and commercially available 3D-printer it is possible to create a simple, cost-effective and high-fidelity 3D-printed model for temporal bone training. ORL surgeons learning the mastoidectomy procedure found the model to be a good supplement to cadaver surgery and rated the replicated anatomical properties of the model to be adequate. This study gives a comprehensive description of technical considerations in 3D-printing of temporal bone models and key points on the production process. With the presented workflow, it is possible for ORL surgeons, without prior experience on 3D-printing, to manufacture their own models for basic temporal bone training. As the model does not accurately replicate finer anatomical structures, training beyond the basic mastoidectomy level or larger scale productions likely requires more *advanced* and costly print technologies.

## Supplementary Information

**Additional file 1.**

## Data Availability

Available on request.
