# Supplementary material for: 3D-printing a cost-effective model for mastoidectomy training
Source: 3D Print Med. 2023 Apr 17;9:12. doi: 10.1186/s41205-023-00174-y (PMC10108487; doi:10.1186/s41205-023-00174-y)
Supplement: Supplementary file 1 — Additional file 1. [file 41205_2023_174_MOESM1_ESM.docx]

**Additional file**

All the materials had the same overall problem: They melted during drilling and shavings was not comparable to real bone. The material with less melting, creating less shavings, most realistic color and with the best tactile feeling was the Laybrick. The following filaments where tested:

1. Wood-filled PLA
2. Metal-filled PLA
3. Copper-filled PLA
4. Concrete-filled PLA
5. PVA +
6. Nylon filament
7. White ABS
8. Laybrick
